# The Impact of Frailty on Palliative Care Receipt, Emergency Room Visits and Hospital Deaths in Cancer Patients: A Registry-Based Study

**DOI:** 10.3390/curroncol30070486

**Published:** 2023-07-11

**Authors:** Peter Strang, Torbjörn Schultz

**Affiliations:** 1 Department of Oncology-Pathology, Karolinska Institutet, Stockholms Sjukhem Foundation, Mariebergsgatan 22, SE 112 19 Stockholm, Sweden; 2 Research and Development Department, Stockholm’s Sjukhem Foundation, Mariebergsgatan 22, SE 112 19 Stockholm, Sweden; torbjornschultz@gmail.com

**Keywords:** cancer, frailty, palliative care services, emergency service, place of death, cancer care transitions

## Abstract

Background. Eastern Cooperative Oncology Group (ECOG) performance status is used in decision-making to identify fragile patients, despite the development of new and possibly more reliable measures. This study aimed to examine the impact of frailty on end-of-life healthcare utilization in deceased cancer patients. Method. Hospital Frailty Risk Scores (HFRS) were calculated based on 109 weighted International Classification of Diseases 10th revision (ICD-10) diagnoses, and HFRS was related to (a) receipt of specialized palliative care, (b) unplanned emergency room (ER) visits during the last month of life, and (c) acute hospital deaths. Results. A total of 20,431 deceased cancer patients in ordinary accommodations were studied (nursing home residents were excluded). Frailty, as defined by the HFRS, was more common in men than in women (42% vs. 38%, *p* < 0.001) and in people residing in less affluent residential areas (42% vs. 39%, *p* < 0.001). Patients with frailty were older (74.1 years vs. 70.4 years, *p* < 0.001). They received specialized palliative care (SPC) less often (76% vs. 81%, *p* < 0.001) but had more unplanned ER visits (50% vs. 35%, *p* < 0.001), and died more often in acute hospital settings (22% vs. 15%, *p* < 0.001). In multiple logistic regression models, the odds ratio (OR) was higher for frail people concerning ER visits (OR 1.81 (1.71–1.92), *p* < 0.001) and hospital deaths (OR 1.66 (1.51–1.81), *p* < 0.001), also in adjusted models, when controlled for age, sex, socioeconomic status at the area level, and for receipt of SPC. Conclusion. Frailty, as measured by the HFRS, significantly affects end-of-life cancer patients and should be considered in oncologic decision-making.

## 1. Introduction

Most oncologists have experienced patients who have unexpectedly developed severe side effects, deteriorated, and even died after standard cancer treatment in an elderly, previously stable patient cohort with acceptable Eastern Cooperative Oncology Group Performance Scores (ECOG PS) and Karnovsky Performance Scores (KPS).

It is well known in oncology that elderly patients might have poorer treatment outcomes and an increased risk of side effects. Therefore, oncological regimens often have set age limits, and ECOG PS and/or KPS are used to identify suitable patients and to avoid treatment in patients in the risk zone [1,2]. Although widely used, the ECOG PS also has limitations [3] as it is a unidimensional functional score and is dependent on assessments by the physician in charge [2]. Moreover, it is seldom used to assess toxicity [4], and its usefulness in modern immunotherapy is less understood [5]. 

For the above-mentioned reasons, additional assessments are needed in modern oncology, especially for older cancer patients, as the discriminative ability of the ECOG PS has its shortcomings; however, assessments are also needed in palliative care situations. Frailty has emerged as a useful measure of vulnerability in the elderly in general [6,7,8], as well as in older cancer patients in need of cancer surgery, medical treatment, or radiotherapy [9,10]. 

Frailty is a wide and complex concept characterized by a decline in physiological capacity across several organ systems and is often defined as an age-related condition with increased vulnerability to acute endogenous exogenous stressors [6,7,11]. As long as there are no external stressors, such as oncological treatments, a person may seem to be in a stable physical and mental condition; however, frail individuals have limited reserve capacity, and their homeostasis is fragile. Therefore, standard chemotherapy treatment may result in physical deterioration as well as acute delirium, rendering a previously independent person dependent on others for their activities of daily living (ADL) [6]. 

Already more than twenty years ago, Fried et al. described the frailty phenotype, which includes five important criteria: low physical activity, weakness (impaired grip strength), poor endurance, slowness (e.g., walking speed), and weight loss [12]. If three of the five criteria are met, a person is characterized as frail. The introduction of the frailty phenotype is important in routine clinical work; however, this phenotype does not describe the underlying pathophysiology that results in the frail phenotype. In fact, frailty is not merely a question of physical weakness, as it manifests itself in several organ systems: a frail brain, a frail immune system, also with alterations in the inflammatory system, a frail hormonal system, as well as a frail muscular metabolism [6]. 

Reduced production of anabolic hormones, an altered immune system, and impaired muscle metabolism may explain physical deterioration after exposure to an external stressor, and a frail brain may explain the increased risk of acute delirium, which is regularly seen in treatment situations.

Several instruments are available for prospective frailty assessments, of which the Clinical Frailty Scale and Frailty Phenotype are the most commonly used tools in acute care settings [13,14]. There are also other clinically useful tools, but these are not always interchangeable, as they differ in their constructs; thus, they differ in length and in their ability to predict mortality, depending on the context [13]. For research purposes, an obvious disadvantage is that these instruments require systematically registered prospective measurements. To overcome this problem, the International Classification of Diseases 10th revision (ICD-10)-based frailty scores have been developed. The Hospital Frailty Risk Score (HFRS) is a validated measure based on 109 weighted ICD-10 diagnoses regularly seen in frail individuals [15]. In a recent study, frailty, as measured by the HFRS, was strongly associated with coronavirus disease-19 (COVID-19) deaths [16].

The proposed frailty definitions, as well as most of the instruments, focus on the physical and biomedical aspects of frailty. This has been criticized, as physical frailty is influenced and promoted by social factors, as reviewed in an article by Gobbens et al. [17]. Social frailty is described as a construct that includes domains such as social needs (e.g., our need for social and emotional connection), resources (a sufficient income, healthy food, accommodation that is adapted to one’s individual needs, medical care, etc.), social fulfillment (engagement and activities), and self-management (cognitive function, mental health, and advance planning) [18]. A person living in social isolation is more likely to develop frailty, and a person with frailty has a higher risk for mental illness; in fact, frailty has even been associated with late-life suicidality [19].

In oncology, frailty has attracted increasing attention in recent years. In cancer patients, frailty is seen in 40–50% of older individuals [9], with far-reaching consequences. Recent reviews and meta-analyses show that frailty in cancer is associated with treatment complications, non-completion of planned treatments, hospitalization, and poor overall survival [9,20,21,22,23,24,25,26]. Considering these outcomes, frail individuals with advanced cancer are likely to have substantial care needs that are preferably offered in Specialized Palliative Care (SPC) settings. 

In the Stockholm Region, with nearly 2.3 million inhabitants, basic palliative care is supposed to be offered at any healthcare level, including primary care, hospital care, or nursing home care. However, people with complex palliative care needs are offered SPC either through advanced palliative home care teams or in palliative inward settings. Typically, oncological treatment and specialized palliative home care are integrated for patients with advanced cancer and complex symptoms; oncological units manage cancer treatments, whereas the home care team provides symptom control, treatment of infections, and monitoring of blood samples. Both home care teams and palliative wards are manned by multi-professional teams, including physicians, registered nurses, physiotherapists, dieticians, and occupational therapists, who operate 24/7, offering successful symptom control and support that is appreciated by both patients and families [27].

Although increasing evidence highlights the importance of frailty in oncological decision-making, little is known about its possible impact on palliative care situations. For this reason, the aim was to characterize cancer patients who died with frailty, as measured by the HFRS, to study frailty in relation to healthcare utilization during the last three months of life, with special reference to the receipt of SPC, Emergency Room (ER) visits, and hospital deaths. 

## 2. Materials and Methods

In this study, we used the Strengthening the Reporting of Observational Studies in Epidemiology (STROBE) criteria in order to increase readability [28].

### 2.1. Study Design

In this retrospective registry study, we analyzed data from all patients who died of advanced cancer between 2015 and 2021 in Stockholm County (with approximately 2.3 million inhabitants).

### 2.2. Population

Inclusion criteria: Patients were eligible if they were aged > 18 years and died with advanced cancer, defined by a primary ICD-10 diagnosis of cancer and a concomitant secondary IDC-10-based diagnosis of distant metastases. Patients with malignant brain tumors or hematological malignancies as the main diagnosis were also included, as distant metastases do not exist in these malignancies.

Exclusion criteria: People with advanced cancer who were nursing home residents were excluded, as most of them are frail by definition and thus not representative of the major group of cancer patients. In Swedish healthcare, elderly people are encouraged to live in their own homes, when needed with the aid of home help services. When a person can no longer manage their daily living despite up to eight home help visits, including night visits, they are considered frail and offered a residency in a nursing home.

### 2.3. Variables

SPC receipt in the last three months of life, unplanned ER visits in the last month of life, and death in an acute hospital were chosen as dependent variables (outcomes). HFRS, age, sex, and socioeconomic Mosaic groups were chosen as independent variables.

SPC: In Region Stockholm, the SPC is designed for patients with excessive and complex needs for pain management, general symptom control, and psychosocial and existential support, and approximately 80% of all enrolled patients are diagnosed with advanced cancer. Patients not suited for advanced home care are offered a bed in a palliative care ward for various reasons. As a rule, each SPC offers both advanced home and inpatient care with its own palliative care department. Both outpatient and inpatient teams are multi-professional, including doctors, nurses, occupational therapists, physiotherapists, and dietitians, and work 24/7 [27].

HFRS: As described in the Introduction, HFRS was developed by Gilbert et al. to identify older, frail individuals at risk of adverse health outcomes using frailty-related ICD-10 diagnoses rather than prospective frailty assessments [15]. To this end, Gilbert et al. used a developmental cohort of over 20,000 patients with significant care needs and multiple frailty-associated diagnoses. A preliminary HFRS score was developed and prospectively tested in over one million patients, including cancer patients, resulting in a validated HFRS score. The final score is based on 109 weighted ICD diagnoses, and patients with values ≥ 5 are labeled as frail.

Mosaic: Mosaic is a commercial socioeconomic measure at the area level to which the Stockholm Region subscribes. It is a system that divides a county or city into small, homogeneous socioeconomic areas [29,30,31]. Thus, Stockholm County is divided into 1300 small areas, each classified as Mosaic 1, 2, or 3, and the groups are approximately the same size. Mosaic Group 1 corresponds to the most affluent areas. Mosaic is based on classical socioeconomic variables such as education and income, but it is also constructed from more than 40 additional variables such as living arrangements, lifestyle, and cultural aspects. The variables were grouped using iterative cluster analyses to form three area-based Mosaic groups, 1–3. In our final analysis, we merged Groups 1 and 2 (i.e., affluent and middle-class areas), which we compared with Mosaic 3 (less affluent area).

### 2.4. Selection Bias and Dropouts

Patients included in the Stockholm Region’s administrative databases used public healthcare services at least once during the study period. The limited group of patients who used private care was also included, as private care providers have financial agreements with the Stockholm Region. Our data are primarily complete, as each clinic and care unit are obliged to report every healthcare visit (primary care, hospital care visits, and hospitalizations) to the database. 

Financial compensation for care is based on reporting; therefore, very few values were missing (estimated to be much less than 1%).

### 2.5. Study Size

No power calculations were performed, as all eligible patients with advanced cancer between 2015 and 2021 were included.

### 2.6. Statistical Methods and Missing Data

We used chi-square tests to compare proportions, and *t*-tests were applied for the comparison of mean values that were normally distributed. In analyses of data that were not normally distributed, the *t*-test was substituted with Mann–Whitney U-test (Wilcoxon rank sum test). Regarding relevant variables, univariable logistic regression models were performed, followed by multivariable logistic regression models. The dataset was complete except for the Mosaic classifications of 175 patients. These missing values were not substituted. For the analyses, SAS 9.4/Enterprise Guide 8.2 (SAS institute, NC 27513-2414, USA) was used.

### 2.7. Ethics

This study was approved by the Regional Ethical Review Authority (Regionala Etikprövningsnämnden i Stockholm 2017/1141-31).

## 3. Results

### 3.1. Clinical Characteristics

A total of 20,431 deceased cancer patients in ordinary accommodations were studied (nursing home residents were excluded) in relation to frailty, as measured by the HFRS (cutoff: an HFRS score of 5 or more). According to the HFRS scores, 12,276 (60%) and 8155 (40%) patients were classified as non-frail and frail, respectively. Those with frailty were older (74.1 versus 70.4 years, *p* < 0.001) and lived more often in less advantaged socioeconomic areas (Mosaic group 3) (42% vs. 39%, *p* < 0.001). Frailty was more common in men than in women (42% vs. 38%, *p* < 0.001). Those with frailty had less often received SPC (76% vs. 81%, *p* < 0.001), and they died more often in acute hospital settings (22% vs. 15%, *p* < 0.001) (Table 1).

### 3.2. Receipt of Palliative Care in Relation to Frailty

Receipt of SPC, i.e., specialized palliative care provided by multi-professional teams 24/7, either in the form of home care teams or as care in a palliative ward, was less common in those with frailty (76% vs. 81%, *p* < 0.001) (Table 1). As frailty was more common in the elderly, in men, and people from less advantaged socioeconomic groups in univariable comparisons (see Table 1), a multivariable logistic regression model was used, where frailty was adjusted for the three mentioned variables, with adjusted Odds Ratios (aOR) Table 2.

After adjusting for age, sex, and socioeconomic Mosaic groups, people with frailty, as measured by HFRS, were still less likely to receive SPC with an aOR of 0.78 (0.72–0.83, *p* < 0.001). Table 2 shows that receipt of SPC was less likely in people who were 80 years or older, who were male, or who belonged to Mosaic group 3 (i.e., a less advantaged group).

### 3.3. Emergency Room (ER) Visits during the Last Month of Life

In total, 8341 (40.8%) individuals made at least one unplanned ER visit during the last month of life. Frail patients had significantly more visits than non-frail cancer patients (50.1% vs. 34.7%, *p* < 0.001) (Table 1).

Also, in an adjusted multiple regression model, frailty was strongly associated with unplanned ER visits with an aOR of 1.81 (1.71–1.92, *p* < 0.001) when adjusted for age, sex, Mosaic group, and receipt of SPC. Receipt of SPC was strongly associated with fewer ER visits, with an aOR of 0.30 (0.28–0.32). Being male, belonging to the oldest age group or a less advantaged Mosaic group (group 3), were weakly associated with more ER visits, with aORs between 1.08–1.13. As shown in Table 3, in a model including HFRS, age had a limited predictive value.

In an additional adjusted multiple regression model, where SPC was excluded from the calculations, the impact of HFRS was unchanged, with an aOR of 1.84 (1.74–1.95, *p* < 0.001). Also, the other variables (age, sex, and Mosaic) were mainly unchanged.

### 3.4. Acute Hospitals as Places of Death

Only a minority of all cancer patients, 3696 (18%), died in an acute hospital setting, with somewhat more hospital deaths for individuals classified as frail compared with non-frail (22% vs. 15%, *p* < 0.001) (Table 1). Of the patients who received SPC, 1043/16,162 (6.4%) died in acute hospitals, whereas the corresponding figure for those who were not enrolled in the SPC was 2653/4269 (62.2%) (*p* < 0.001).

In a multiple regression model adjusted for age, sex, socioeconomic area (Mosaic), and receipt of SPC, frailty (HFRS) was the only variable that was significantly associated with a higher likelihood of hospital deaths, aOR 1.66 (1.51–1.81, *p* < 0.001) (Table 4). Moreover, there was an extremely strong inverse relationship between the receipt of SPC and hospital deaths–people receiving SPC were very unlikely to die in an acute hospital setting, OR 0.04 (0.036–0.043, *p* < 0.001). Compared with patients aged 18–64 years, elderly patients were less likely to die in acute care hospitals (Table 4). Sex and Mosaic groups were not statistically associated with hospital deaths in the adjusted model.

## 4. Discussion

Our aim was to characterize cancer patients who died with frailty, as measured by the HFRS, and to study frailty in relation to healthcare utilization at the end of life. We also wanted to focus on the effect of SPC on acute healthcare consumption. Although all patients died with advanced cancer, there were still differences between those classified as frail and those who were not.

In this cohort of deceased cancer patients, frailty was found more often in older cancer patients, in men, and among people living in less advantaged socioeconomic areas (Mosaic 3). Moreover, they received SPC to a lesser extent but had more unplanned ER visits and acute hospital deaths. The proportion of patients defined as frail in our study was 40%, consistent with other frailty studies on cancer [9].

The association between age and frailty was expected, as there is consensus that, in most cases, frailty develops as a consequence of age-related decline in multiple physiological systems [6]. Simultaneously, there is a common understanding that chronological age cannot be translated directly into biological age.

In oncology, age-related matters are of clinical interest, as cancer incidence and prevalence increase with increasing life expectancy. Cancer patients today are older, and previous age limits in treatment guidelines need to be reconsidered. Currently, biological and functional heterogeneity among patients of the same age is mainly assessed using the ECOG PS and KPS to support treatment decisions [1,2]. Although simple and robust, both performance scores have shortcomings in their discriminative ability. Therefore, geriatric screening tools such as the G8 have been tested in geriatric cancer populations [32], and recently, Takahashi et al. suggested such geriatric assessments of frailty to support decision-making [33]. E.g., Miller et al. showed that frailty might be a better predictor than age in terms of outcomes and complications after rectal cancer surgery [34], well in line with a recent review of colorectal cancer surgery, where 15 studies were included (almost 100,000 patients) [35], and also in line with a meta-analysis on cancer surgery at large [36].

Frailty assessments have also been used to assess the risk of toxicity during chemotherapy [10,37,38]. In fact, the National Cancer Institute suggests that geriatric assessments of frailty should be included in future clinical trials to better characterize the trial population, define eligibility, to predict outcomes, and, eventually, personalize cancer treatments [39].

Sometimes, multimorbidity/comorbidity and frailty are considered the same condition, but frailty with an increased risk of adverse outcomes can occur in the absence of evident comorbidity [6]. Therefore, there is a clinical need to screen for frailty in elderly patients with cancer, not only considering diagnosed comorbidities.

In our study, frailty was observed more often in men and in people living in less affluent socioeconomic areas. This is of interest, as there are relatively few data on frailty in relation to sex in different settings. In a study of community-dwelling people, frailty was more prevalent among women [40], as in a Taiwanese population-based survey that was not adjusted for confounding factors such as age [41]. However, such findings cannot be directly transferred to the context of cancer, as the cancer population is not randomly drawn from the general population. As observed during the COVID-19 pandemic, the adjusted mortality rates were consistently higher among men and among people with cancer [16]. Frailty was also seen more often in people from less socioeconomically advantaged neighborhoods, consistent with previous studies [42,43].

Patients with cancer and frailty had a lower aOR for receiving SPC during the last three months of life. At first glance, this is surprising, as frail people are expected to require more help than non-frail people. This finding could not be explained by our registry study. However, a possible explanation could be that we have a large proportion of patients in need of SPC but also a subset of patients in whom symptoms of frailty predominate, giving rise to a greater need for help with activities of daily living (ADL), rather than a need for help with complex cancer symptoms. In the Swedish healthcare system, daily assistance with ADL is provided by the municipal home help service, and medical support to the elderly is provided by primary care or by geriatric units as long as the needs are related to chronic non-cancer comorbidities, whereas SPC is reserved for cancer patients with intense cancer-related symptoms and complex care needs.

Concerning unplanned ER visits during the last month of life, frail cancer patients had a significantly higher aOR of 1.81, indicating an approximately 81% higher probability of ER visits. This association also persisted when the receipt of SPC was excluded from the adjusted multivariable model. This is an important finding, as unplanned and burdensome ER visits are often seen as markers of reduced quality of life in dying people [44].

Moreover, hospital deaths were more common among frail patients with cancer (aOR, 1.66). In addition to frailty as measured by HFRS, the likelihood of dying in acute hospitals was highly associated with the receipt of SPC; the figures for those who received versus did not receive SPC were 6% and 62%, showing a remarkable difference but well in line with our data on specific cancer forms, such as breast, prostate, or lung cancer [45,46]. Our findings are also in line with our data for patients with severe chronic obstructive pulmonary disease (COPD) and heart failure [46,47]. Dying in an acute hospital is not always avoidable, but it is not an optimal place for death, as hospitals are not designed for the palliative care of an acutely dying person. In fact, acute hospital settings are the least preferred place of death for patients [48].

### 4.1. Strengths and Limitations

Generally, frailty studies have been conducted in limited groups, as most frailty studies are based on prospective, time-consuming measurements, sometimes with missing values. Therefore, the study results can only be transferred to similar contexts. Our registry data, with exceptionally few missing values and with frailty data based on 109 weighted ICD-10 codes that are regularly seen in frail people, provides a comprehensive picture of the impact of frailty, which is a strength. Moreover, most studies are based on samples, which affects representativeness. This study is based on the total cohorts from 7 years, from a reasonably large area (catchment area of 2.3 million inhabitants), which strengthens the results regarding reliability and validity. Data can mainly be generalized to similar, tax-funded healthcare systems.

One limitation is that we did not have access to death certificates and relied on ICD-10-based primary and secondary diagnoses for care episodes at the end of life. Thus, in the individual case, we cannot know whether the immediate cause of death was cancer in itself or a common complication (such as pulmonary embolism), but we do know that these patients died with a clinical picture of advanced cancer. Moreover, we used the ICD-10-based HFRS score to define frailty. Other measures, e.g., prospective measures with the Clinical Frailty Score, could have influenced the results to some extent.

### 4.2. Future Research

As previously mentioned, the National Cancer Institute already suggests that geriatric assessments of frailty should be included in future clinical trials [39]. However, by using HFRS, it is also possible to re-evaluate previously completed studies, especially in cases where adverse effects were unexpectedly severe or where the response was less positive than expected, as such outcomes may be related to people with high frailty scores. Such knowledge could be used to better define eligibility criteria in future clinical trials. Moreover, baseline frailty assessments can be used as a basis for preventive geriatric interventions in connection to chemotherapy treatment, with promising results on completion of therapy, toxicity, and quality of life [49], but more studies are needed.

## 5. Conclusions

In conclusion, the heterogeneity concerning frailty is considerable, also in a group of patients who died of advanced cancer. Moreover, although frailty is strongly related to age, age and frailty do not go hand in hand. Instead, frailty scores help to better define biological age.

Higher frailty scores were independently associated with a higher need for unplanned ER visits and higher probability of hospital deaths, also when adjusted for age, which should be considered for care planning and dimensioning of care.

## Figures and Tables

**Table 1:** Characteristics of patients aged > 18 years who died with advanced cancer ^a^ (*n* = 20,431) in Stockholm County from 2015 to 2021, with or without frailty, as measured by HFRS.

Variable	Total *n* = 20,431	Non-Frail *n* = 12,276	Frail *n* = 8155	*p*-Value ^b^
**Age**, all, years, mean (SD)	71.9 (12.2)	70.4 (12.2)	74.1 (11.8)	<0.001
**Sex**				<0.001 ^c^
Women, *n* (%)	10,021 (49)	3824 (61.8)	6197 (38.2)	
Men, *n* (%)	10,410 (51)	6079 (58.4)	4331 (41.6)	
**Mosaic groups**				<0.001 ^d^
Groups 1 + 2, *n* (%)	13,859 (67.8)	8478 (61.2)	5381 (38.8)	
Group 3, *n* (%)	6572 (32.2)	3798 (57.8)	2774 (42.2)	
**Admitted to SPC ^e^**, *n* (%)	16,162 (79.1)	9956 (81.1)	6206 (76.1)	<0.001
**ER visits ^f^**, *n* (%)	8341 (40.8)	4259 (34.7)	4082 (50.1)	<0.001
**Hospital deaths**, *n* (%)	3696 (18.1)	1880 (15.3)	1816 (22.3)	<0.001

^a^ Advanced cancer: patients with distant metastases, diagnosis of CNS, or hematological malignancy. ^b^ *p*-values between those who were classified as non-frail or frail, respectively. ^c^ chi-2 comparison between women and men. ^d^ chi-2 comparison between Mosaic groups 1 + 2, versus group 3. ^e^ SPC: Specialized palliative care via home care or care in a palliative care ward. ^f^ At least one ER visit during the last month of life.

**Table 2:** Adjusted Odds ratios (aORs) for the probability of receiving SPC (specialized palliative care). *n* = 20,431 cases of advanced cancer ^a^, of which 16,162 received SPC in the last three months of life.

Variable	Adjusted Odds Ratio aOR (95% CI)	*p*-Value
**Frailty** groups ^b^		
Non-frail (HFRS < 5)	Ref.	
Frail (HFRS ≥ 5)	0.78 (0.72–0.83)	<0.001
**Age groups**		
18–64 years	Ref.	
65–79 years	1.02 (0.94–1.12)	0.57
≥80 years	0.78 (0.71–0.86)	<0.001
**Sex**		
Female	Ref.	
Male	0.75 (0.70–0.80)	<0.001
**Mosaic socioeconomic groups**		
1 + 2 (advantaged groups)		
3 (less advantaged group)	0.88 (0.82–0.94).	<0.001

^a^ Advanced cancer: patients with distant metastases or a diagnosis of CNS or hematological malignancy. ^b^ Hospital Frailty Risk Score (HFRS).

**Table 3:** Adjusted Odds ratios (aORs) for the probability of unplanned ER visits during the last month of life. *n* = 20,431 cases of advanced cancer ^a^, of which 8341 had at least one ER visit.

Variable	Adjusted Odds Ratio aOR (95% CI)	*p*-Value
**Frailty** groups ^b^		
Non-frail (HFRS < 5)	Ref.	
Frail (HFRS > 5)	1.81 (1.71–1.92)	<0.001
**Receipt of SPC**		
No	Ref.	
Yes	0.30 (0.28–0.32)	<0.001
**Age groups**		
18–64 years	Ref.	
65–79 years	1.00 (0.93–1.08)	0.96
≥80 years	1.10 (1.01–1.19)	0.03
**Sex**		
Female	Ref.	
Male	1.08 (1.02–1.14)	0.01
**Mosaic socioeconomic groups**		
1 + 2 (advantaged groups)	Ref.	
3 (less advantaged group)	1.13 (1.06–1.20)	<0.001

^a^ Advanced cancer: patients with distant metastases or a diagnosis of a CNS tumor or hematological malignancy. ^b^ Hospital Frailty Risk Score (HFRS).

**Table 4:** Adjusted Odds ratios (aORs) for the probability of having acute hospitals as a place of death. *n* = 20,431 cases of advanced cancer ^a^, of which 3696 died in an acute hospital setting.

Variable	Adjusted Odds Ratio aOR (95% CI)	*p*-Value
**Frailty** groups ^b^		
Non-frail (HFRS < 5)	Ref.	
Frail (HFRS > 5)	1.66 (1.51–1.81)	<0.001
**Receipt of SPC**		
No	Ref.	
Yes	0.04 (0.036–0.043)	<0.001
**Age groups**		
18–64 years	Ref.	
65–79 years	0.85 (0.76–0.95)	0.04
≥80 years	0.51 (0.44–0.58)	<0.001
**Sex**		
Female	Ref.	
Male	1.03 (0.99–1.12)	0.56
**Mosaic socioeconomic groups**		
1 + 2 (advantaged groups)	Ref.	
3 (less advantaged group)	1.00 (0.91–1.10)	0.97

^a^ Advanced cancer: patients with distant metastases or a diagnosis of a CNS tumor or hematological malignancy. ^b^ Hospital Frailty Risk Score (HFRS).

## Data Availability

The datasets generated and analyzed in this study are available upon reasonable request.
